# Aroclor 1254 inhibits anti-inflammatory macrophage polarization through an AhR-dependent mechanism

**DOI:** 10.1210/jendso/bvaf205

**Published:** 2025-12-16

**Authors:** Riley M Behan-Bush, Elizabeth Kilburg, Jesse N Liszewski, Michael V Schrodt, Edward A Sander, Aloysius J Klingelhutz, James A Ankrum

**Affiliations:** Roy J. Carver Department of Biomedical Engineering, University of Iowa, Iowa City, IA 52242, USA; Fraternal Order of Eagles Diabetes Research Center, University of Iowa, Iowa City, IA 52242, USA; Roy J. Carver Department of Biomedical Engineering, University of Iowa, Iowa City, IA 52242, USA; Roy J. Carver Department of Biomedical Engineering, University of Iowa, Iowa City, IA 52242, USA; Fraternal Order of Eagles Diabetes Research Center, University of Iowa, Iowa City, IA 52242, USA; Roy J. Carver Department of Biomedical Engineering, University of Iowa, Iowa City, IA 52242, USA; Fraternal Order of Eagles Diabetes Research Center, University of Iowa, Iowa City, IA 52242, USA; Roy J. Carver Department of Biomedical Engineering, University of Iowa, Iowa City, IA 52242, USA; Fraternal Order of Eagles Diabetes Research Center, University of Iowa, Iowa City, IA 52242, USA; Department of Microbiology and Immunology, University of Iowa, Iowa City, IA 52242, USA; Roy J. Carver Department of Biomedical Engineering, University of Iowa, Iowa City, IA 52242, USA; Fraternal Order of Eagles Diabetes Research Center, University of Iowa, Iowa City, IA 52242, USA

**Keywords:** PCB, polychlorinated biphenyl, toxicology, adipose, metabolism, PCB126

## Abstract

Macrophages are critical regulators of tissue homeostasis and inflammation. During the development of chronic inflammatory diseases, tissue-resident macrophages often shift from an anti-inflammatory (M2) to a pro-inflammatory (M1) phenotype. Understanding the factors that drive this polarization shift is essential for elucidating the mechanisms underlying diseases such as cancer, cardiovascular disease, and metabolic syndrome. Environmental toxicants, including polychlorinated biphenyls (PCBs), may be key contributors to this dysregulation. Despite being banned in the United States for nearly 50 years, PCBs persist in the built and natural environment, with mixtures such as Aroclor 1254 still detected at concerning levels in schools and other public spaces. In this study, we investigated how Aroclor 1254 influences human monocyte-derived macrophage polarization. We found that exposure to Aroclor 1254 during differentiation skews naïve macrophages toward a pro-inflammatory phenotype, enhances LPS/IFNγ-driven M1 polarization, and inhibits both IL-4- and dexamethasone-induced M2 polarization. To explore underlying mechanisms, we examined the roles of peroxisome proliferator-activated receptor gamma (PPARγ), pyruvate kinase M2 (PKM2), and the aryl hydrocarbon receptor (AhR). We found AhR inhibition partially rescued PCB-mediated suppression of M2 polarization. Further supporting this mechanism, PCB126, a potent AhR agonist, recapitulated the disruption of polarization. Together, these findings demonstrate that PCB mixtures act through AhR to dysregulate macrophage polarization, driving a pro-inflammatory phenotype. This disruption may represent a key mechanism by which PCBs exacerbate tissue inflammation and contribute to the pathogenesis of chronic inflammatory diseases.

As part of the innate immune system, macrophages are essential for host defense against pathogens and cancers (1). They reside in virtually all tissues, poised to recognize and eliminate invading microbes as they appear. However, macrophages are far more than simple phagocytes. They are highly versatile cells that contribute to tissue homeostasis and influence disease processes across the body (2). For instance, macrophages regulate bone density, recycle alveolar surfactant in the lungs, clear senescent erythrocytes in the liver, provide metabolic support to neurons, and maintain the metabolic and endocrine functions of adipose tissue (3). Given these wide-ranging roles, understanding the macrophage niche is central to elucidating the pathogenesis of many diseases.

One reason macrophages can perform such diverse functions is their remarkable phenotypic plasticity (4, 5). Unlike T-cells that undergo terminal differentiation, macrophages remain highly plastic, continuously responding to their microenvironment (6). In healthy tissues, macrophages typically adopt an anti-inflammatory, M2-like phenotype. Within this category, subtypes such as M2a macrophages promote wound healing, while M2c macrophages facilitate the resolution of inflammation. In contrast, when tissues experience injury, infection, cancer, or other inflammatory insults, macrophages often shift toward a pro-inflammatory, M1-like phenotype. This transition promotes the recruitment of circulating monocytes, which differentiate into additional inflammatory macrophages, amplifying inflammation within the tissue (4). Although this M2-to-M1 transition is a normal component of the immune response, persistent environmental or cellular stressors can sustain macrophage activation and monocyte recruitment, driving a cycle of chronic inflammation. Chronic inflammation is a defining feature of many disorders, including cardiovascular disease, cancer, autoimmune disease, and metabolic syndrome (7). Thus, identifying the factors that promote inflammatory macrophage phenotypes is critical for understanding the origins of chronic inflammation.

One such factor that could contribute to chronic inflammation is exposure to environmental toxicants. Of particular interest, due to their ubiquitous presence in our built environment, is polychlorinated biphenyls, or PCBs. PCBs are a group of toxicants made up of 209 distinct congeners. They were originally manufactured by Monsanto as bulk mixtures of congeners and sold under the tradename *Aroclor* (8). Valued for their non-flammability and insulating properties, Aroclor mixtures were widely incorporated into products such as paints, caulks, light ballasts, electrical transformers, and many other common materials (9-12). Although manufacturing was banned in the United States in 1979, Aroclor mixtures remain persistent in the environment and are frequently detected at elevated levels in older buildings, including schools constructed in the 20th century. In fact, the high school in Burlington, VT was just demolished in 2024 due to the detection of unacceptably high levels of PCBs throughout the school. In studies published as recent as 2022, signatures of Aroclor 1254 continue to be measured at high levels in school air (9, 13, 14). Importantly, teachers and students in these environments show elevated serum PCB levels that follow the patterns of Aroclor 1254 exposure, underscoring ongoing human exposure even 40 years after PCB production was banned (15).

Epidemiological studies link PCB exposure to increased incidence of chronic inflammatory metabolic diseases, including diabetes and obesity (16-20), yet the mechanistic pathways underlying these associations remain unclear. Since we know that lipophilic PCBs will accumulate in adipose depots over time, one hypothesis is that PCBs impair metabolic health through disruption of adipose tissue (21-23). Since accumulation of fat mass through adipocyte expansion is an important part of the adipose tissue disease process, early work, including our own, focused on elucidating the impacts of PCBs on adipocyte expansion and adipogenesis. Our group demonstrated that Aroclor 1254 augments adipogenesis in adipose-derived mesenchymal stem cells (MSCs), leading to adipocyte hypertrophy (24). However, the magnitude of PCB effects on MSCs was modest, prompting the exploration as to whether other adipose-resident cell types, particularly macrophages, are more sensitive to PCB toxicity.

To date, few studies have examined PCB effects on macrophages. Animal studies suggest that PCB exposure alters the adipose immune environment, increasing macrophage infiltration and systemic inflammatory mediator release, including interleukin-6 (IL-6) and tumor necrosis factor (TNF) (25, 26). However, this in vivo work has not identified whether it is PCB-mediated macrophage dysfunction that results in greater inflammation. There have been only a few studies that have looked at the direct impacts of PCBs on macrophages. In vitro studies exposing macrophages to single PCB congeners have revealed that dioxin-like (DL) PCBs, such as PCB126 and PCB118, enhance M1 polarization, while non-dioxin-like (NDL) congeners, such as PCB153, may exert anti-inflammatory effects by dampening lipopolysaccharide (LPS) responses (27-29). However, these studies do not reflect real-world exposures, which involve complex mixtures of congeners. Because PCBs were manufactured as Aroclor mixtures, humans are exposed to dozens of congeners simultaneously, potentially leading to interactions that alter toxicity profiles. Our prior work demonstrated that already polarized M2 macrophages exposed to Aroclor 1254, a mixture containing both DL and NDL congeners, will undergo phenotypic plasticity toward a pro-inflammatory phenotype (30). However, plasticity of already polarized macrophages is just one mechanism by which PCBs could alter the immune balance. Another mechanism is by impacting the recruitment, differentiation, and polarization of monocytes into macrophages. Unfortunately, little is known about how PCB accumulation in adipose tissue impacts the initial polarization of macrophages.

To fill this gap, we investigated how PCB exposure influences macrophage polarization using primary human monocyte-derived macrophages, a model with high translational relevance. We first assessed the effects of Aroclor 1254 exposure on naïve macrophages, then examined its impact on M1 vs M2 polarization. After discovering substantial impact of Aroclor 1254 exposure on macrophage polarization, we explored potential mechanisms of disruption by probing the roles of peroxisome proliferator-activated receptor gamma (PPARγ), pyruvate kinase M2 (PKM2), and the aryl hydrocarbon receptor (AhR).

## Methods

### Sources of PCBs

Stocks of Aroclor 1254 (Lot #: KC 12-638) and 3,3′,4,4′,5-pentachlorobiphenyl (PCB126) were obtained from the Synthesis Core of Iowa Superfund Research Program (ISRP) at University of Iowa (31). Each PCB stock was dissolved in 100% dimethyl sulfoxide (DMSO) before use in cell culture experiments.

### Cell culture media

During monocyte-to-macrophage differentiation and polarization, cells were cultured in RPMI 1640 medium (Gibco, Cat#: 11875093) supplemented with 10% (v/v) fetal bovine serum (FBS; VWR, Cat#: 97068-085), 1% (v/v) penicillin-streptomycin (Thermo Fisher, Cat#: 15140122), and 1% (v/v) L-glutamine (Thermo Fisher, Cat#: 25030081). For experiments involving PCB treatment, a low-serum version of RPMI 1640 containing 0.5% (v/v) FBS was utilized.

### Isolation of human PBMCs

Leukocyte reduction cones (LRCs) from human blood donors were provided by the DeGowin Blood Center at the University of Iowa (IRB#201103721). Upon receipt, blood was flushed from the LRCs using base RPMI and layered onto LeucoSep tubes prefilled with Ficoll-Paque (Cytiva, Cat#: 17544202). The samples were centrifuged at 600*g* for 30 minutes without brake. The resulting buffy coat layer was harvested and washed twice with phosphate-buffered saline (PBS) containing 2% FBS. Red blood cells were eliminated using 1× RBC lysis buffer (Cytek, Cat#: TNB-4300-L100), followed by an additional wash in complete RPMI. The isolated peripheral blood mononuclear cells (PBMCs) were then counted and prepared for subsequent monocyte isolation. PBMCs from 16 individual donors were used throughout the study, with the number of donors per experiment specified in the corresponding figure legends. The sex and age for each donor can be found in supplemental information (32).

### Isolation of monocytes

The monocytes were isolated from PBMCs using the MojoSort Human Pan Monocyte Isolation Kit (BioLegend, Cat#: 480060), which is a magnetic bead-based negative selection isolation kit. PBMCs were first washed in 1× MojoSort Buffer (PBS supplemented with 0.5% BSA and 2mM EDTA) and passed through a 40-μm cell strainer. The cell concentration was adjusted to 1 × 10⁸ PBMCs/mL in MojoSort Buffer. Briefly, Fc receptors were blocked were incubating cells in the biotin-antibody cocktail and magnetic streptavidin nanobeads. The samples were then washed and resuspended MojoSort Buffer. Once prepared, tubes were placed in a MojoSort™ magnet (BioLegend, Cat#: 480019) for 5 minutes, after which the supernatant containing the enriched monocyte population was collected. The bead-bound fraction was rewashed, and magnetic separation was repeated to maximize recovery. Monocytes were cryopreserved at 1 × 10⁷ cells/mL in a freezing solution of 50% complete RPMI, 40% FBS, and 10% DMSO. For experiments, frozen monocytes were thawed at 37 °C and transferred into culture medium.

### Monocyte-to-macrophage differentiation

Thawed monocytes were resuspended at a concentration of 1 × 10⁶ cells/mL in culture medium supplemented with macrophage colony-stimulating factor (M-CSF; 10 ng/mL, BioLegend, Cat#: 574806) to promote differentiation. Cells were plated in 24-well tissue culture plates at a density of approximately 4 × 10⁵ cells/cm², corresponding to 750 K cells/well in 750 μL. Differentiation proceeded over a 6-day period with a media change on day 3. On day 6, the naïve (M0) macrophages were ready for PCB exposure and polarization.

### Macrophage PCB exposure and polarization

Once differentiated, the naïve macrophages were exposed to either PCBs or the vehicle control (VC), DMSO (0.5 μL/mL), for 24 hours in low-serum media. After this initial exposure in low-serum media, the media was replaced with normal serum culture media supplemented again with PCBs or DMSO as well as the components for M1, M2a, or M2c polarization, as defined previously, for 48 hours (33-35). For M1 polarization, the media was supplemented with 50 ng/mL of IFNγ (PeproTech, Cat#: 300-02) and 25 ng/mL of LPS from Escherichia coli O55:B5 (Sigma Aldrich, Cat#: L6529). For M2a polarization, the media was supplemented with 20 ng/mL IL-4 (PeproTech, Cat#: 200-04). For M2c polarization, the media was supplemented with 100nM dexamethasone (Sigma Aldrich, Cat#: D4902).

To assess whether inhibition of the aryl hydrocarbon receptor (AhR) alters the effects of PCB exposure, macrophages were pretreated with the AhR antagonist CH223191 (8μM; Sigma Aldrich, Cat#: C8124) for 2 hours to achieve receptor blockade. Following this pretreatment, PCB exposure and macrophage polarization were carried out as described previously. In the AhR-inhibited groups, CH223191 was maintained at a concentration of 4μM throughout the duration of PCB exposure and polarization.

### Surface marker expression

Flow cytometry was used to evaluate macrophage surface marker expression. To preserve surface antigens, adherent macrophages were detached on ice using 5mM EDTA in PBS (36). Macrophages were first stained for viability using the Zombie B550 Fixable Viability Kit (BioLegend, Cat#: 423122). To minimize nonspecific binding, True-Stain Monocyte Blocker (BioLegend, Cat#: 426103) and Human TruStain FcX (BioLegend, Cat#: 422302) were applied. Surface staining was then performed using the following anti-human antibodies: CD14-Alexa Fluor 488 (BioLegend, Cat#: 325610, RRID:AB_830683), CD16-PE-Fire 640 (BioLegend, Cat#: 302068, RRID:AB_2876587), CD86-PerCP-Cy5.5 (BioLegend, Cat#: 374216, RRID:AB_2734432), CD163-PE-Cy7 (BioLegend, Cat#: 333614, RRID:AB_2562641), and CD206-PE-Dazzle 594 (BioLegend, Cat#: 321130, RRID:AB_2616867). Stained cells were washed and resuspended in Cell Staining Buffer (BioLegend, Cat#: 420201) prior to acquisition.

Flow cytometry was conducted using a Cytek Northern Lights spectral analyzer equipped with a 488 nm laser and 14-channel emission detection. Instrument setup and gating strategies were established using unstained cells and polarized macrophages as controls. Data were analyzed in FlowJo (BD Sciences), with gating based on singlets (Forward Scatter Area/Height (FSC-A/H) and Side Scatter Area/Height (SSC-A/H)) and viability (exclusion of Zombie B550+ cells). Surface marker levels were reported as median fluorescence intensity (MFI). To confirm proper M1, M2a, and M2c polarization, we compared our experimental surface marker expression to expected expression from previous works (33).

### Enzyme-linked immunosorbent assays

To measure the release of inflammatory or anti-inflammatory cytokine release, the amount of IL-8 and IL-10 in culture media was quantified using enzyme-linked immunosorbent assays (ELISA; IL-8: BioLegend, Cat#: 431504; IL-10: BioLegend, Cat#: 430604) (37). Culture supernatants were harvested at the conclusion of each experiment and stored at −20 °C until analysis. ELISAs were carried out according to the manufacturer's instructions. Absorbance readings were obtained at 450 nm, and background correction was performed by subtracting absorbance at 570 nm. Standard curves generated using known concentrations of IL-8 and IL-10 served as positive controls, and assay diluent was used as a negative control.

### SCENITH assay

The levels of cellular metabolism were measured using the SCENITH (Single-Cell ENergetIc metabolism by Translational Inhibition) assay (38, 39). For each experimental replicate, 3 conditions were prepared in parallel wells, each receiving a distinct metabolic inhibitor or control. At the start of the assay, culture media was removed and adherent macrophages were gently rinsed once with cold PBS. Cells were then treated for 20 minutes at 37 °C with one of the following: 2-deoxyglucose (DG; 100mM), the negative control harringtonine (2 μg/mL), or complete RPMI as a positive control. Following this pretreatment, puromycin (10 μg/mL) was added directly to the wells, and cells were incubated for an additional 30 to 45 minutes at 37 °C. After incubation, all media and reagents were removed, and cells were washed once more with cold PBS.

Macrophages were then detached using 5mM EDTA in PBS on ice to preserve surface markers. After collection, cells were washed and stained with Zombie B550 Fixable Viability Dye (BioLegend, Cat#: 423122) to identify live cells. Fc receptor blocking was performed simultaneously using Human TruStain FcX™ (BioLegend, Cat#: 422302). After a 15-minute room temperature incubation, cells were washed in PBS and fixed with FluoroFix Buffer (BioLegend, Cat#: 422101) for 30 minutes. Permeabilization was carried out using Intracellular Staining Permeabilization Wash Buffer (BioLegend, Cat#: 421002), followed by intracellular staining with PE-conjugated anti-puromycin antibody (BioLegend, Cat#: 381504, RRID:AB_2927816) for 1 hour at 4 °C. Finally, stained cells were washed twice and analyzed by flow cytometry.

Flow cytometric data was processed using FlowJo software (BD Biosciences). Gating strategy included singlet discrimination (Forward Scatter Area vs Height (FSC-A/H) and Side Scatter Area vs Height (SSC-A/H)), followed by selection of live cells (Zombie B550-negative). A baseline gate was established using the harringtonine control, and puromycin incorporation was quantified based on PE fluorescence using median fluorescence intensity (MFI) as the readout. Glucose dependence was calculated as described by Arguello and colleagues (38).

### Quantitative polymerase chain reaction

To extract macrophage RNA, TRIzol/chloroform extraction was performed. After phase separation, the clear aqueous phase was collected. RNA clean-up was performed using the Zymo RNA Clean & Concentrator-5 kit (Zymo, Cat#: R1013) and isolated into 15 μL of nuclease-free water. The amount of RNA isolated from each sample, as well as protein and solvent purity, was quantified using nano-drop. The RNA was converted into cDNA using a reverse transcriptase kit (Applied Biosystems; Cat #4375575). Applied Biosystems QuantStudio 7 Flex was used for quantitative polymerase chain reactions (qPCR) with SYBR green master mix (Applied Biosystems; Cat #4367659). The chosen primers can be found in Table 1. Cyclophilin A (PPIA) was chosen for gene normalization and gene changes were reported as −ΔCt = −(Ct_TARGET_ − Ct_housekeeping_).

**Table 1:** qRT-PCR primers

Primer name; exon location	IDT catalog #
PPIA	58v.38887593.g
PPARG; 7-8	58.25464465
PKM; 4-5b	58.14798218

### Data analysis

For experimental outcome, a total of 3 to 5 independent experiments were done with monocyte-derived macrophages from separate, unique human donors. For each donor/independent experiment, each exposure condition (VC/PCB) was performed in parallel in triplicate wells. The mean of the triplicate wells was then quantified and used as a single data point to represent the average for that donor. The donors used within each figure can be found in supplemental information (32).

All statistical analyses and data visualization were conducted using GraphPad Prism version 10. All analysis accounted for the monocyte donor (paired *t* tests, and repeated measures analysis of variance [ANOVA]) used for each of the unique experiments. Statistical significance was defined as *P* < .05. Additional details regarding statistical testing and sample size are provided in the corresponding figure legends.

## Results

### PCB exposure alone shifts naïve macrophages toward an inflammatory phenotype

**Figure 1:**
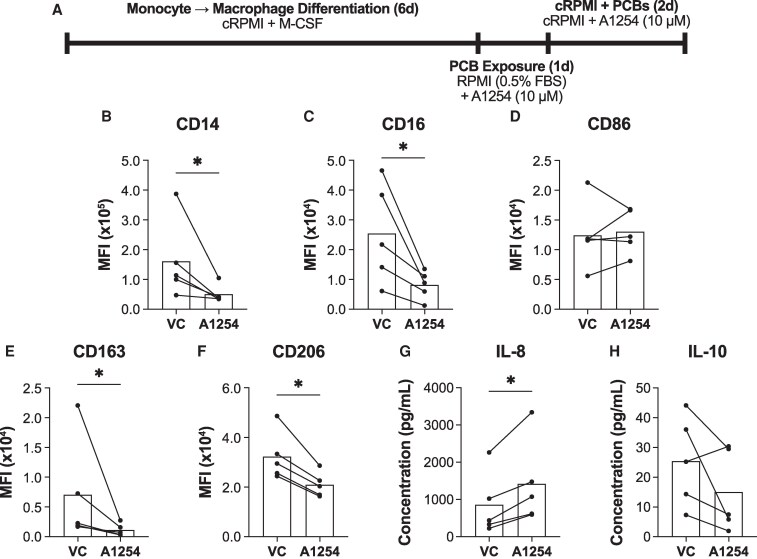
PCB exposure pushes naïve macrophages toward a more inflammatory phenotype. (A) Naïve monocyte-derived macrophages were exposed to Aroclor 1254 (10μM) or the vehicle control (VC) DMSO for 3 days. Surface markers were analyzed using flow cytometry after which the median fluorescent intensity (MFI) was quantified for (B) CD14, (C) CD16, (D) CD86, (E) CD163, and (F) CD206. Donors used for surface marker experiments include 23.06, 23.07, 23.08, 23.09, 24.02. The concentrations of (G) IL-8 and (H) IL-10 were measured via ELISA. Donors used for cytokine experiments include 23.02, 19.16, 21.1, 19.14, 23.08. (mean, n = 5 human donors/independent experiments, df = 4, *P < .05 calculated after paired *t* test).

Building on our previous findings that Aroclor 1254 exposure induces macrophage plasticity toward a more inflammatory phenotype, we next sought to determine whether this exposure also affects initial macrophage polarization. To investigate this, we assessed whether PCB exposure alone could polarize naïve macrophages toward either an inflammatory or anti-inflammatory phenotype. We exposed naïve human monocyte-derived macrophages to a 10μM concentration of Aroclor 1254 for 3 days (1 day in low serum, followed by 2 days in normal serum RPMI) (Fig. 1A). This concentration of Aroclor 1254 was chosen to mimic the exposure macrophages would have in adipose tissue. Depending on the severity of PCB exposure, adipose tissue can contain anywhere from 700 to 9000 ng/g lipid of total PCBs. Considering the molecular weight of PCBs as well as the density and volume of lipid within adipose tissue, this concentration corresponds to a range of 1.5μM to 18μM of total PCB (23, 24, 40). After the macrophages were exposed to PCBs, we evaluated phenotypic changes by measuring surface marker expression and cytokine release.

We found that PCB exposure reduced the expression of general macrophage markers, CD14 (*P* < .01) and CD16 (*P* < .01) (Fig. 1B and 1C). Additionally, exposure preferentially downregulated the anti-inflammatory markers CD163 (*P* < .01) and CD206 (*P* < .01), while leaving the expression of the inflammatory marker CD86 (*P* = .44) unchanged (Fig. 1D and 1F). These findings suggest that PCB exposure shifts macrophages away from an anti-inflammatory phenotype. Further support for this conclusion came from the analysis of cytokine release. Although PCB exposure caused a nonsignificant decrease in the anti-inflammatory cytokine IL-10 (*P* = .08), it resulted in a 2-fold increase in IL-8 (*P* < .01), a pro-inflammatory marker (Fig. 1G and 1H). Together, these results indicate that PCB exposure alone promotes macrophage polarization toward a more inflammatory phenotype.

### PCB exposure enhances inflammatory, M1 polarization

**Figure 2:**
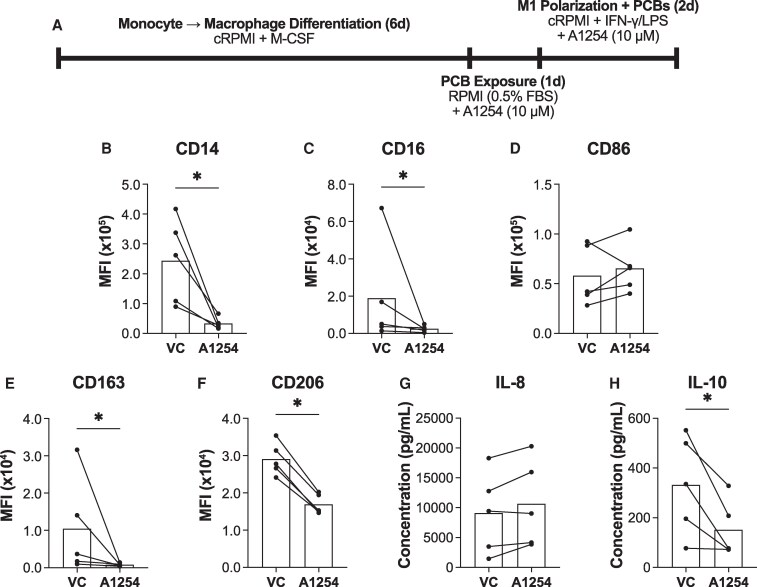
PCB exposure enhanced M1 polarization. (A) Timeline of macrophage differentiation, M1 polarization, and exposure to Aroclor 1254 (10μM) or the vehicle control (VC) DMSO. Surface markers were analyzed using flow cytometry after which the median fluorescent intensity (MFI) was quantified for (B) CD14, (C) CD16, (D) CD86, (E) CD163, and (F) CD206. Donors used for surface marker experiments include 23.06, 23.07, 23.08, 23.09, 24.02. The concentrations of (G) IL-8 and (H) IL-10 were measured via ELISA. Donors used for cytokine experiments include 23.02, 19.16, 21.1, 19.14, 23.08. (mean, n = 5 human donors/independent experiments, df = 4, *P < .05 calculated after paired *t* test).

Macrophage polarization is influenced by their surrounding environment, and it is unlikely that macrophages will be exposed to PCBs in isolation, without other stimuli. Therefore, we next investigated how PCB exposure affects macrophage polarization. To begin, we examined whether PCB exposure enhances inflammatory macrophage polarization. We exposed naïve macrophages to PCBs in low serum for 24 hours, then polarized them toward an inflammatory M1 phenotype using IFNγ and LPS in the presence of PCBs (Fig. 2A).

Macrophages polarized to the M1 phenotype in the presence of PCBs displayed enhanced polarization toward an inflammatory phenotype. Compared to the vehicle-exposed cells, PCB-exposed cells showed decreased expression of CD14 (*P* < .01) and CD16 (*P* = .03) (Fig. 2B and 2C). Additionally, these M1 macrophages exhibited reductions in the expression of anti-inflammatory markers CD163 (*P* = .02) and CD206 (*P* < .01) (Fig. 2E and 2F). However, similar to the naïve macrophages in Fig. 1, there was no change in CD86 (*P* = .28) expression (Fig. 2D). Cytokine analysis revealed that PCB exposure halved IL-10 (*P* = .03) secretion (Fig. 2H). However, there was little change in IL-8 (*P* = .18) secretion, which may be due to the macrophages reaching the peak of their IL-8 release capacity (Fig. 2G). Together, these results demonstrate that PCB exposure enhances M1 polarization in macrophages.

### PCB exposure inhibits anti-inflammatory M2 polarization

**Figure 3:**
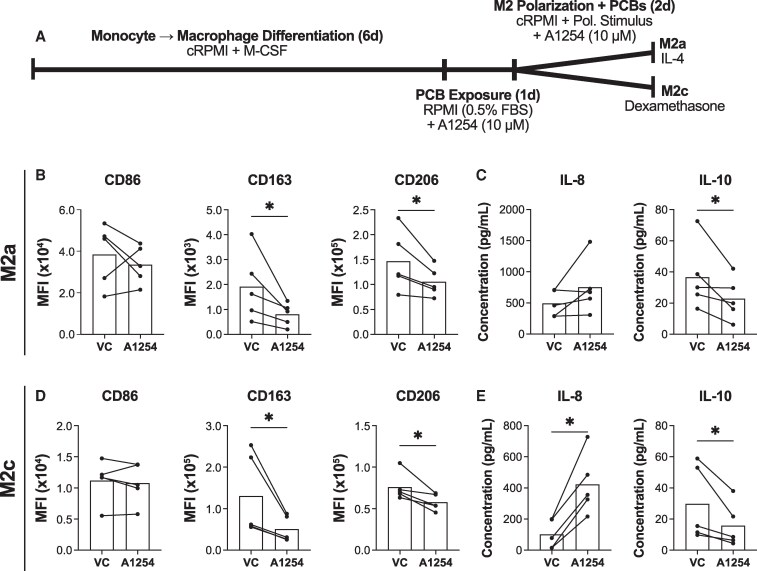
PCB exposure inhibits M2 polarization. (A) Timeline of macrophage differentiation, M2a/M2c polarization, and exposure to Aroclor 1254 (10μM) or the vehicle control (VC) DMSO. Cells were exposed to Aroclor 1254 or the vehicle control throughout polarization before quantifying surface markers (CD86, CD163, and CD206) using flow cytometry (median fluorescent intensity (MFI)) and cytokines (IL-8 and IL-10) via ELISA for (B, C) M2a and (D, E) M2c cells. Donors used for surface marker experiments include 23.06, 23.07, 23.08, 23.09, 24.02. Donors used for cytokine experiments include 23.02, 19.16, 21.1, 19.14, 23.08. (mean, n = 5 human donors/independent experiments, df = 4, *P < .05 calculated after paired *t* test).

Having shown PCBs enhance the inflammatory phenotype both in the absence of stimuli and when an inflammatory stimulus is present, we next assessed whether PCB exposure inhibits the polarization of anti-inflammatory macrophage phenotypes. Given the diversity of anti-inflammatory macrophage phenotypes in vivo, we focused on 2 types of M2 subtypes: M2a macrophages polarized with IL-4 and M2c macrophages polarized with dexamethasone. Naïve macrophages were exposed to PCBs for 24 hours before being polarized toward the M2a or M2c phenotype in the presence of PCBs (Fig. 3A). As before, macrophage polarization was assessed using surface marker expression and cytokine release.

Both M2a and M2c macrophage polarization were profoundly impacted by PCB exposure. Similar to the naïve and M1 macrophages, both M2a and M2c macrophages exposed to PCBs showed decreased expression of CD14 (M2a: *P* < .01, M2c: *P* < .01) and CD16 (M2a: *P* < .01, M2c: *P* = .08) (Supplemental Data) (32). These PCB-exposed M2 macrophages also exhibited preferential downregulation of the anti-inflammatory surface markers CD163 (M2a: *P* < .01, M2c: *P* < .01) and CD206 (M2a: *P* = .01, M2c: *P* = .01), while expression of CD86 persisted (M2a: *P* = .60, M2c: *P* = .55) (Fig. 3B and 3D). For cytokine release, both M2a and M2c macrophages showed an increase in IL-8 (M2a: *P* = .12, M2c: *P* = .01), although this was only statistically significant in the M2c macrophages. Both phenotypes also exhibited a significant decrease in the release of the anti-inflammatory cytokine IL-10 (M2a: *P* = .04, M2c: *P* < .01) (Fig. 3C and 3E). Altogether, Figs. 1 to 3 show that PCB exposure alters macrophage polarization, driving them toward a more inflammatory phenotype.

### PCBs do not inhibit M2 polarization through a PPARγ or PKM2 mechanism

**Figure 4:**
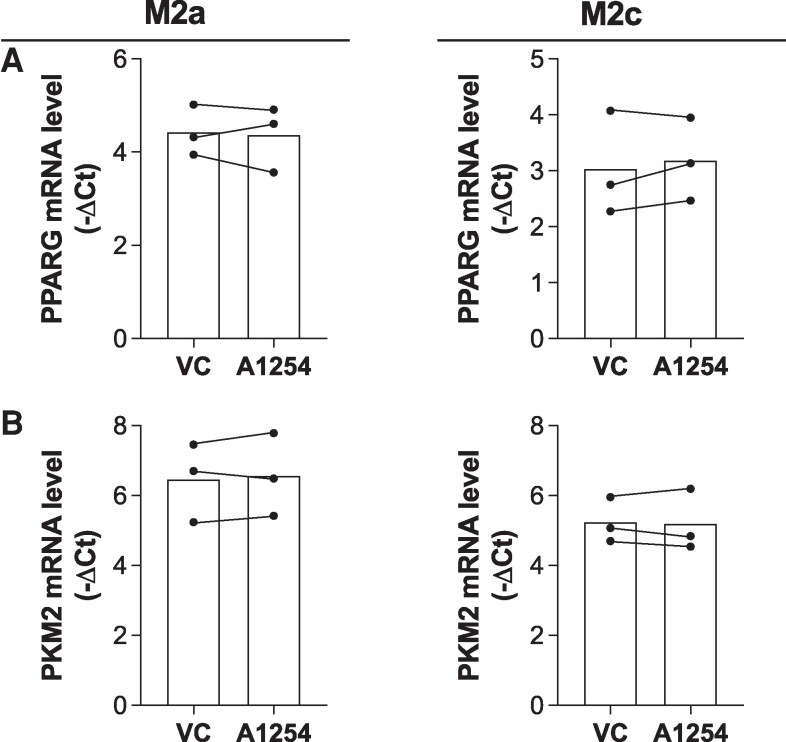
PCB exposure does not alter PPARG or PKM2 gene expression. M2a and M2c macrophages were exposed to Aroclor 1254 (10μM) or the vehicle control (VC) DMSO throughout polarization. mRNA levels for (A) PPARG and (B) PKM2 were quantified using RT-qPCR followed by −ΔCt analysis compared to the housekeeping gene cyclophilin A (−ΔCt = −(Ct_TARGET_ − Ct_housekeeping_)). Donors used for PPARG experiments include 25.03, 25.02, 25.05. Donors used for PKM2 experiments include 25.02, 25.05, 25.06 (mean ± SD, n = 3 human donors/independent experiments, df = 2, *P < .05 calculated after paired *t* test).

Having shown that Aroclor 1254 enhances inflammatory macrophage polarization and inhibits anti-inflammatory polarization, we next sought to investigate potential mechanisms underlying these effects. First, we examined whether PPARγ or PKM2 signaling plays a role in this dysfunction. PPARγ (ie, peroxisome proliferator-activated receptor gamma) is a transcription factor that regulates the polarization and differentiation of various cells. Although PPARγ is most commonly associated with adipocyte differentiation, it also contributes to alternative, M2 macrophage polarization (41, 42). Given that we have previously shown that Aroclor 1254 reduces *PPARG* mRNA expression, inhibiting adipogenesis in adipose mesenchymal stromal cells (MSCs) (24), we hypothesized that Aroclor 1254 could be acting through PPARγ in macrophages. To test this hypothesis, macrophages were exposed to Aroclor 1254 and polarized toward an M2a or M2c state as previously described. After the 48 hours of polarization, mRNA was isolated from the macrophages, and transcript levels of *PPARG* were analyzed via RT-qPCR. Interestingly, Aroclor 1254 exposure did not result in gene expression changes of *PPARG* (M2a: *P* = .84, M2c: *P* = .40), revealing that changes in PPARγ gene expression are not responsible for PCB-induced dysfunction of macrophage polarization (Fig. 4A).

The next mechanism we evaluated was pyruvate kinase M2 (PKM2), a glycolytic enzyme that links metabolic dysfunction to inflammation in macrophages. During inflammatory activation, glycolytic flux increases, and PKM2 drives the transcription of inflammatory genes, promoting an M1-like phenotype (43, 44). Since we had previously shown that Aroclor 1254 enhances glycolytic flux in macrophages exposed after polarization (30), we hypothesized there would be changes in *PKM2* gene expression alongside the changes in phenotype we observed. Yet again, Aroclor 1254 exposure did not result in changes of *PKM2* gene expression (M2a: *P* = .53, M2c: *P* = .80) (Fig. 4B). Thus, we did not detect evidence of PCB-induced M2 polarization dysfunction being mediated through alterations in the gene expression of PPARγ or PKM2.

### Blocking the aryl hydrocarbon receptor partially prevents PCB effects on M2 polarization

We next assessed the role of the aryl hydrocarbon receptor (AhR) in PCB-induced disruption of macrophage polarization. AhR functions primarily as an environmental sensor, particularly for bacterial and environmental contaminants. Depending on the ligand that binds to AhR, it can either promote pro-inflammatory or anti-inflammatory macrophage phenotypes. In the case of 2,3,7,8-tetrachlorodibenzo-p-dioxin (TCDD) and other DL compounds, activation of AhR is known to promote a pro-inflammatory response via nongenomic pathways (45). Since Aroclor 1254 contains many DL PCB congeners, we hypothesized that AhR activation contributes to the enhanced inflammatory profile we have observed.

**Figure 5:**
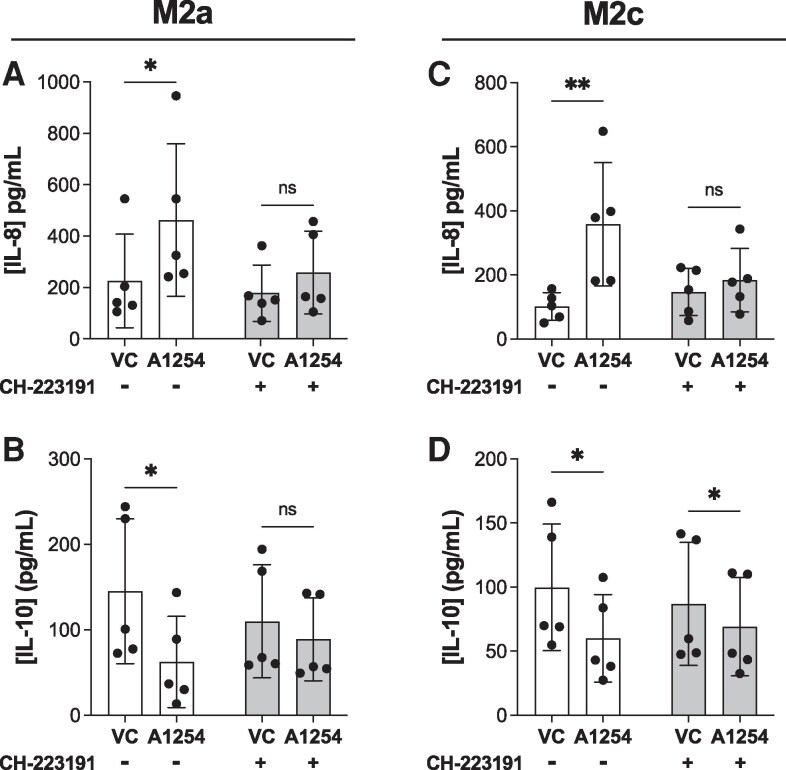
AhR blockade partially prevents PCB-induced inhibition of M2 polarization. M2a macrophages were polarized with or without Aroclor 1254 (10μM) exposure (vehicle control [VC] = DMSO) and with or without the AhR antagonist CH-223191. Then cytokine levels of (A) IL-8 and (B) IL-10 were measured using ELISA. M2c macrophages were polarized with or without Aroclor 1254 (10μM) exposure (VC = DMSO) and with or without the AhR antagonist CH-223191. Then cytokine levels of (A) IL-8 and (B) IL-10 were measured using ELISA. Donors used include 25.03, 25.02 (2 independent experiments), 25.05, 25.06 (mean ± SD, n = 5 human donors/independent experiments, 2-way ANOVA with matching by human donor, DFn = 1, DFd = 4, *P < .05 on Šídák multiple comparisons test, comparisons were made between VC and A1254 within each AhR block condition).

To investigate the role of AhR signaling, we blocked AhR using the antagonist CH-223191 throughout the duration of PCB exposure and polarization. The effects of the blockade were assessed by measuring IL-8 and IL-10 secretion. We found that the PCB-induced disruption of both M2a and M2c macrophages was prevented by blocking the AhR receptor. As expected, M2a macrophages exposed to PCBs during polarization exhibited a statistically significant increase in IL-8 and a decrease in IL-10. However, when CH-223191, an AhR inhibitor, was present throughout the experiment, there was no significant difference between the PCB- and vehicle-exposed groups (Fig. 5A and 5B). This was further supported by 2-way ANOVA, where both PCB exposure (IL-8: *P* = .048, IL-10: *P* = .021) and the interaction between PCB exposure and AhR blockade (IL-8: *P* = .016, IL-10: *P* = .009) were statistically significant (*P* < .05), while AhR blockade alone did not show a significant effect on either IL-8 or IL-10 (IL-8: *P* = .077, IL-10: *P* = .609).

A similar result was observed for M2c macrophages. PCB-exposed M2c macrophages showed a statistically significant increase in IL-8 and a decrease in IL-10. However, with AhR blockade, the impact of PCB exposure on IL-8 expression was inhibited, while the decrease in IL-10 was less pronounced (Fig. 5C and 5D). Again, 2-way ANOVA confirmed that both PCB exposure (IL-8: *P* = .041, IL-10: *P* = .007) and the interaction between PCB exposure and AhR blockade (IL-8: *P* = .015, IL-10: *P* = .005) were statistically significant (*P* < .05), while AhR blockade alone did not reach statistical significance (IL-8: *P* = .053, IL-10: *P* = .702). Overall, these data demonstrate that blocking AhR partially prevents Aroclor 1254 from inhibiting anti-inflammatory polarization in macrophages.

### AhR agonist PCB126 recapitulates inhibition of M2 polarization in a dose-dependent manner

Next, we wanted to see if we could replicate the effects of the PCB mixture Aroclor 1254 by exposing macrophages to a single dioxin-like PCB congener. PCB126 is the most potent dioxin-like congener and is known to mediate its toxicities through AhR signaling (46-49). Therefore, we aimed to determine whether PCB126 exposure also results in inhibition of M2 polarization. To do this, we performed the same exposure and polarization protocol used with Aroclor 1254, but instead exposed the macrophages to PCB126 at concentrations of 4nM, 200nM, or 10μM. Given that PCB126 is a strong activator of AhR, the dose response was chosen to explore the effects at varying concentrations.

**Figure 6:**
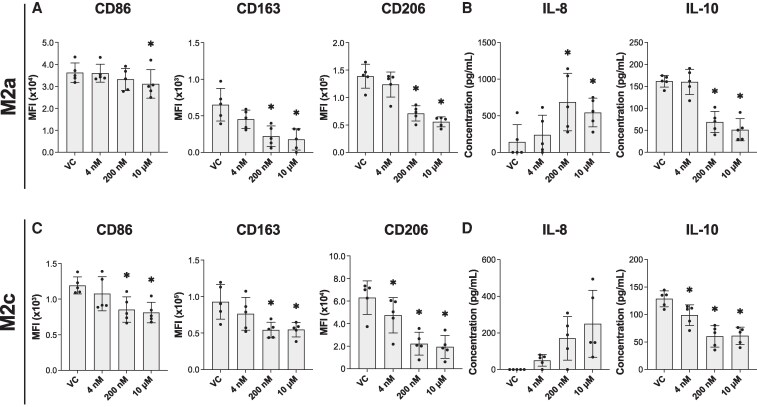
AhR agonist PCB126 results in a dose-dependent inhibition of M2 polarization. M2a macrophages were polarized in the presence of PCB126 (4nM, 200nM, or 10μM) or the vehicle control (VC) DMSO before quantifying (A) surface markers (CD86, CD163, and CD206) using flow cytometry (median fluorescent intensity [MFI] and (B) cytokines (IL-8 and IL-10) via ELISA. M2c macrophages were polarized in the presence of PCB126 (4nM, 200nM, or 10μM) or the vehicle control (VC) DMSO before quantifying (A) surface markers (CD86, CD163, and CD206) using flow cytometry (MFI) and (B) cytokines (IL-8 and IL-10) via ELISA. Donors used include 25.05, 25.06, 25.07, 23.09, 24.06 (mean ± SD, n = 5 human donors/independent experiments, df = 19, 1-way ANOVA with matching by human donor, *P < .05 compared to VC-treated cells on Dunnett's multiple comparisons test).

Our results show that PCB126 inhibits M2a polarization in a manner similar to Aroclor 1254. Upon analysis with one-way ANOVA, PCB126 exposure caused a dose-dependent decrease in the anti-inflammatory surface markers CD163 (*P* < .01) and CD206 (*P* < .01), while the expression of CD86 (*P* = .02) only had a significant downward shift at the 10μM concentration (Fig. 6A). Additionally, M2a macrophages exhibited a dose-dependent increase in IL-8 (*P* < .01) and a decrease in IL-10 (*P* < .01) upon PCB126 exposure (Fig. 6B).

PCB126-exposed M2c macrophages showed a similar pattern. Upon analysis with one-way ANOVA, the expression of both pro-inflammatory (CD86 (*P* < .01)) and anti-inflammatory (CD163 (*P* = .02) and CD206 (*P* < .01)) markers decreased in a dose-dependent manner with PCB exposure, although the magnitude of change in the anti-inflammatory markers exceeded that of CD86 (Fig. 6C). Furthermore, PCB126 exposure led to a dose-dependent increase in IL-8 (*P* = .03) and a decrease in IL-10 (*P* < .01) for M2c macrophages (Fig. 6D). Notably, M2c macrophages appeared to be more sensitive to PCB126, as statistically significant changes in CD206 expression and IL-10 release were observed even at the 4nM concentration. Overall, these results strengthen the conclusion that PCB activation of the aryl hydrocarbon receptor leads to the inhibition of anti-inflammatory macrophage polarization.

### Inhibition of AhR prevents PCB-induced increase in glycolysis in M2a but not M2c macrophages

Thus far, we have shown that Aroclor 1254 inhibits anti-inflammatory macrophage polarization and enhances the inflammatory features of macrophages. We have also demonstrated that this shift in surface markers and cytokine secretion occurs through an AhR-dependent mechanism. Next, we assessed the impact of PCBs and AhR blockade on the macrophage immunometabolic profile. It is well documented that an upregulation in glycolytic activity is necessary for the activation of inflammatory M1 macrophages (50). Therefore, our next questions were 2-fold: First, is Aroclor 1254-induced inhibition of M2 polarization accompanied by increased glycolysis? Second, does AhR blockade prevent PCB-induced immunometabolic shifts?

**Figure 7:**
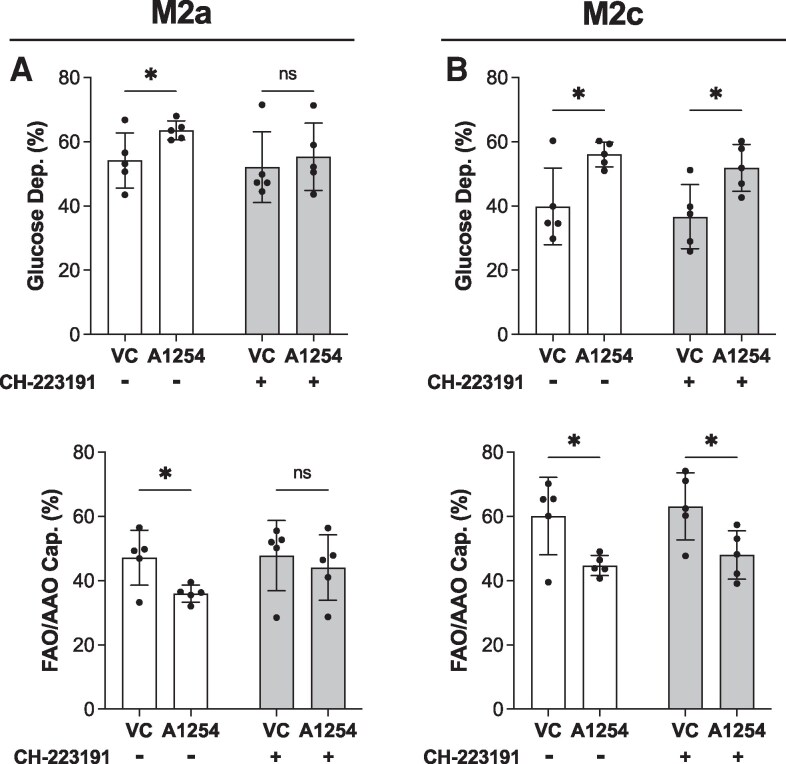
AhR blockade only prevents PCB-induced glycolysis upregulation in M2a macrophages. (A) M2a macrophages were polarized with or without Aroclor 1254 (10μM) exposure (vehicle control VC = DMSO) and with or without the AhR antagonist CH-223191. Then, glucose dependence and fatty acid oxidation (FAO)/amino acid oxidation (AAO) capacity were measured using the SCENITH assay. (B) M2a macrophages were polarized with or without Aroclor 1254 (10μM) exposure (VC = DMSO) and with or without the AhR antagonist CH-223191. Then, glucose dependence and FAO/AAO capacity were measured using the SCENITH assay. Donors used include 25.06, 25.07 (2 independent experiments), 25.05, 25.08 (mean ± SD, n = 5 human donors/independent experiments, 2-way ANOVA with matching by human donor, DFn = 1, DFd = 4, *P < .05 on Šídák multiple comparisons test, comparisons were made between VC and A1254 within each AhR block condition).

As expected, we found that both M2a and M2c macrophages exposed to Aroclor 1254 during polarization had enhanced glucose dependence (M2a: *P* = .03, M2c: *P* < .01), along with decreased fatty acid and amino acid oxidation capacity (M2a: *P* = .01, M2c: *P* < .01). Interestingly, blocking the AhR receptor dampened this effect in M2a glucose dependence (*P* = .42) and fatty acid/amino acid oxidation capacity (*P* = .30) but had no impact on M2c glucose dependence (*P* < .01) and fatty acid/amino acid oxidation capacity (*P* < .01) (Fig. 7A and 7B). Therefore, PCB disruption through the AhR may be dependent on the subtype of M2 macrophage.

## Discussion

Despite the widespread role of macrophages in the development of disease, there has been limited research investigating how PCBs impact this crucial cohort of cells. In this paper, we investigated the impacts of Aroclor 1254, a PCB mixture known to contribute to human exposure, on the polarization of human monocyte-derived macrophages. Our goal was to build on the work of Wang and colleagues, who showed that PCB126 exposure causes naïve macrophages to polarize toward a more inflammatory state and enhances M1 polarization of THP-1 cells (27). We first sought to reproduce these findings with a few changes to reflect a more human-relevant exposure. The first change we made was to use human monocyte-derived macrophages instead of the THP-1 cell line, since in vitro polarization of primary monocyte-derived macrophages is more akin to the in vivo process (51, 52). The second change we made was to expose the macrophages to Aroclor 1254, a PCB mixture, to reflect the complexity of PCB signatures found in humans. Consistent with Wang and colleagues, we found that Aroclor 1254 alone induces inflammatory macrophage polarization and acts synergistically with IFNγ/LPS to enhance the inflammatory polarization of macrophages (Figs. 1 and 2). This is evident from decreased anti-inflammatory surface marker expression (CD163 and CD206), reduced secretion of anti-inflammatory cytokines (IL-10), and increased secretion of inflammatory cytokines (IL-8). We also found decreased expression of general macrophage markers CD14 and CD16 across all macrophage polarization schema (Figs. 1 and 2, and Supplemental Data) (32). CD14 and CD16 are both receptors responsible for enhancing phagocytic clearance, a process incredibly important in tissue healing and the resolution of inflammation (53, 54). Although we did not measure phagocytosis directly, a reduction in these markers further suggests a shift away from the highly phagocytic, M2 state (55). Altogether, it is particularly noteworthy that PCBs amplify the inflammatory response of macrophages, given that toxicant exposure is rarely the sole factor influencing tissue health. In the case of adipose tissue, inflammation is also driven by hypertrophic adipocytes, which release inflammatory mediators in response to excess lipid storage (56, 57). Therefore, PCB accumulation in adipose tissue could happen in the context of preexisting inflammation. These results suggest that PCB exposure in the presence of inflammatory mediators would amplify the M1 polarization of macrophages, leading to paracrine and endocrine effects that could promote further adipose tissue inflammation.

Macrophages are polarized in response to the totality of their surrounding microenvironment. In an already inflammatory environment, our results show that Aroclor 1254 adds an additional inflammatory stimulus. However, what about in the case of a preexisting anti-inflammatory environment that should be present in metabolically healthy individuals? Our previous work found that human monocyte-derived M2 macrophages exposed to Aroclor 1254 underwent phenotypic plasticity toward a more inflammatory phenotype (30). Thus, we hypothesized that Aroclor 1254 would also inhibit or disrupt the initial anti-inflammatory polarization. Our results confirmed this hypothesis, showing that Aroclor 1254 inhibits both IL-4 (M2a) and dexamethasone-stimulated (M2c) macrophage polarization, resulting in reduced anti-inflammatory surface marker expression (CD163) and altered cytokine release (increased IL-8 and decreased IL-10) (Fig. 3). These findings are particularly relevant in the context of childhood PCB exposure. Children are among the most exposed populations due to the prevalence of PCBs in schools and are less likely than adults to have preexisting adipose tissue dysfunction (58, 59). Consequently, PCB exposure occurs in the context of healthy adipose tissue. There is evidence to suggest that elevation in inflammatory immune cells within adipose tissue is an early driver of tissue dysfunction and obesity in children (60-63). Since Aroclor 1254 will prevent proper polarization of anti-inflammatory macrophages, it is possible that PCB exposure could incite improper macrophage balance within adipose tissue, contributing to a higher risk of metabolic dysfunction in otherwise healthy adipose tissue.

To deepen our understanding of PCB-induced macrophage polarization dysfunction, we next evaluated several likely mechanisms that could be driving the inflammatory phenotype. We first looked at PPARγ, a well-known inducer of anti-inflammatory macrophage polarization (41, 42, 64, 65). Surprisingly, Aroclor 1254 did not impact the mRNA expression of *PPARG* (Fig. 4). These results are in contrast to both our previous work, showing that Aroclor 1254 inhibits adipose MSC adipogenesis via PPARγ inhibition (66), and other studies showing that individual PCB congeners can augment *PPARG* expression (67-69). Notably, all of these other studies were in adipocyte lineage cells, suggesting there are cell-type-specific impacts of PCBs. Similarly, we explored whether alterations in PKM2 expression could mediate PCB-induced inflammation. Despite prior studies suggesting that PCBs enhance PKM2 expression in HeLa and SMMC-7721 cells (70, 71), we did not observe these changes with Aroclor 1254 exposure to human macrophages (Fig. 4). There are a few possibilities for the lack of an observed change. First, it is possible that PCBs do not induce transcription changes of PPARγ or PKM2 but instead impact activity through post-translational modifications. Second, it is possible that the lack of changes observed is due to our use of a PCB mixture. Humans are very rarely exposed to single congeners of PCBs (15, 40, 72). Therefore, our choice to investigate Aroclor 1254 was intentionally done to probe how human-relevant PCB exposure impacts macrophage polarization. However, Aroclor 1254 alone contains over 100 distinct PCB congeners (9). Each PCB congener has its own mechanisms of action, meaning the resulting phenotype is a culmination of all PCBs contained within Aroclor 1254. Therefore, the key conclusion from this data is not that PCB congeners do not influence PPARγ or PKM2 at all, but rather that the sum of the PCB congeners in Aroclor 1254 results in no significant gene expression changes for either target in macrophages.

Since Aroclor 1254 contains many PCB congeners, we next sought to determine which class of congeners is primarily responsible for its toxicity. PCB congeners are classified as either dioxin-like (DL) or non-dioxin-like (NDL) (73). The DL PCBs are structurally similar to 2,3,7,8-tetrachlorodibenzo-p-dioxin (TCDD), are coplanar, and activate the aryl hydrocarbon receptor (AhR) to varying degrees (74, 75). In contrast, NDL congeners act through other mechanisms, including activation of ryanodine receptors involved in calcium signaling and endocrine receptors, particularly thyroid hormone receptors (76, 77). Although Aroclor 1254 contains both DL and NDL congeners, its high toxic equivalency factor (TEQ = 21) indicates a substantial DL content (78). We therefore hypothesized that DL congeners drive the observed alterations in macrophage polarization. We found that inhibition of AhR with CH-223191 prevented Aroclor 1254–mediated inhibition of M2 macrophage polarization (Fig. 5). Moreover, exposure to PCB126, a highly potent DL congener, reproduced the macrophage toxicity pattern seen with Aroclor 1254 (Fig. 6). These results parallel those found by Wang and colleagues who showed that PCB126 enhanced M1 polarization through an AhR-dependent mechanism (27). They also corroborate our team's previous findings that showed that PCBs, particularly PCB126, mediate pre-adipocyte toxicity and inflammation through AhR (79-83). Together, there is strong evidence to indicate that DL congeners are to a large degree responsible for the Aroclor 1254-mediated pro-inflammatory shift in macrophages.

To probe deeper into the mechanism and beyond AhR, we finished our analysis by examining changes to the metabolism of macrophages due to exposure to Aroclor 1254. We found Aroclor 1254 increased glucose dependence in M2 macrophages—another hallmark of inflammatory activation—but AhR blockade only prevented this effect in M2a, but not M2c, macrophages (Fig. 7). Altogether, the AhR block data suggest that AhR activation alone does not fully account for the effects of PCBs on macrophage polarization. Several factors may explain why AhR is not solely responsible for PCB-induced macrophage dysfunction. First, NDL congeners could contribute to the pro-inflammatory phenotype. Because Aroclor 1254 contains both DL and NDL congeners, AhR inhibition would block only DL-driven effects, leaving NDL activity intact. There is previous work to suggest that NDL congeners act as antagonists to the glucocorticoid receptor, which would prevent the anti-inflammatory activation of macrophages (84). Since NDL congeners would not be impacted by AhR blockade, this could explain why dexamethasone-exposed M2c macrophages were less impacted by AhR-mediated PCB toxicity. However, counter to our results, NDL congeners have been reported to have anti-inflammatory effects on macrophages in prior reports. For example, Santoro and colleagues showed that PCB 101, 153, and 180, all NDL congeners, inhibited LPS-stimulation of the J774A.1 macrophage cell line (29). Overall, no studies have examined mixtures of NDL congeners, and it is possible that their combined effects differ from those observed in single-congener mechanistic studies.

Second, AhR itself plays a complex role in macrophage polarization. Its downstream signaling varies depending on ligand specificity and can occur via genomic or nongenomic pathways. Prior work has shown that TCDD, the prototypical dioxin, activates AhR signaling in macrophages through a nongenomic pathway (45). The nongenomic pathway is also responsible for AhR-mediated increases in IL-8 secretion (85). However, there has been no unified consensus as to whether AhR upregulates or downregulates glucose metabolism (86). It is therefore plausible that DL PCBs in Aroclor 1254 engage AhR to alter the cytokine secretion and surface marker expression via nongenomic AhR activation, while the PCB-mediated shifts in metabolic state may instead be primarily mediated through the NDL congeners. This argument is further strengthened by prior work showing that NDL congeners such as PCB52 and PCB153 have potent impacts on the metabolic profile of cells (70, 87). Future experiments would need to be done to parse out the contributions of the DL and NDL congeners when exposed to mixtures.

The goal of this study, together with our previous work, was to assess the impact of exposure to a human-relevant PCB mixture on macrophage polarization and plasticity. In both our prior work on plasticity and the current work on polarization, we consistently observed that Aroclor 1254 skews macrophages toward a pro-inflammatory phenotype, regardless of whether exposure occurs during or after polarization. This persistent shift toward an M1-like state has important implications for tissues such as adipose, where increased M1 macrophage abundance is a hallmark of dysfunction. Although PCBs preferentially accumulate in adipose tissue, they also distribute to other organs, including the brain and liver (88, 89). Furthermore, each of these tissues is also greatly impacted by the M1/M2 balance of resident macrophages. In the brain, M2 microglia provide neuroprotection while a shift toward M1 dominance exacerbates neuronal injury and contributes to neurodegenerative diseases (90). Similarly, in the liver, an M1-skewed macrophage population worsens injury and fibrosis (91). Despite the role of macrophages in each of these disease processes, most PCB studies have focused only on toxicity mediated through astrocytes and hepatocytes, often overlooking the indirect mechanism these toxicants can exert by altering the phenotype of resident immune cells. As the field advances, it will be critical to integrate immune cell responses into assessments of environmental toxicant effects.

## Data Availability

Original data generated and analyzed during this study are included in this published article or in the data repositories listed in References (32).

## Abbreviations

AhR: aryl hydrocarbon receptor
ANOVA: analysis of variance
DL: dioxin-like
DMSO: dimethyl sulfoxide
ELISA: enzyme-linked immunosorbent assay
FBS: fetal bovine serum
IFNγ: interferon gamma
IL-6: interleukin 6
LPS: lipopolysaccharide
MSC: mesenchymal stem cell
NDL: non-dioxin-like
PBMC: peripheral blood mononuclear cell
PBS: phosphate-buffered saline
PCB: polychlorinated biphenyl
PKM2: pyruvate kinase M2
PPARγ: peroxisome proliferator activated receptor gamma
qPCR: quantitative polymerase chain reaction
VC: vehicle control
